# Why Retirement and Age Discrimination Policies Need to Consider the Intersectional Experiences of Older Women

**DOI:** 10.1093/geroni/igaa057.2939

**Published:** 2020-12-16

**Authors:** Patrick Button, Theodore Figinski, Joanne Song McLaughlin, Ian Burn

**Affiliations:** 1 Tulane University, New Orleans, Louisiana, United States; 2 United States Department of the Treasury, Washington, District of Columbia, United States; 3 SUNY Buffalo, Buffalo, New York, United States; 4 University of Liverpool, Liverpool, England, United Kingdom

## Abstract

We summarize how older women face intersectional experiences that affect their retirement security. These include differential trends in aging, life expectancy, labor supply, work history, retirement savings, and poverty at old age. We also highlight research showing that older women experience significantly more age discrimination than older men. affecting the ability for older women to improve their retirement security by working longer. We demonstrate through examples that these differential trends and intersectional experiences of older women have important policy implications. We provide examples of how Social Security policies, such as increases in the full benefit retirement age and changes to the retirement earnings test, have differential effects on older women. We also discuss how age and gender employment discrimination law fails to protect older women from intersectional sex-plus-age discrimination. We conclude by urging policymakers to consider how older women experience different challenges and how policy should consider their unique experiences.

